# Tubercular Peritonitis, or Scrofulous Inflammation of the Abdominal Viscera

**Published:** 1865-06

**Authors:** W. H. Triplett

**Affiliations:** Washington, D.C.


### TUBERCULAR PERITONITIS, OR SCROFULOUS INFLAMMATION OF THE ABDOMINAL VISCERA.

By W. H. TRIPLETT, M.D., Washington, D.C.

Communicated for the Boston Medical and Surgical Journal.

This is a chronic disease in the wildest acceptation of the term, and, withal, the most ambiguous. Why medical writers have so generally overlooked it altogether, or made but cursory allusion to it, is a mystery to me, inasmuch as they must have had some of these cases under their charge. Not less than four or five have come under my own observation. They are an eccentric and peculiar class of cases, and, so far as my own experience is concerned, have occasioned me more annoyance and perplexity than would be believed. I shall never forget the trouble my first patient caused me, and though at last I had the satisfaction of knowing that her disease had been incurable from the first, yet had I been fully advised of its nature, I think I might have withheld much useless medicine, and been spared many moments of needless anxiety.

When I opened the abdomen of the dead woman, the mystery was at once explained. I was amazed at the organic lesions that were revealed: bands of solidified lymph, in various stages of organization, fastened and glued the abdominal organs in a confused and matted mass, putrid with fœtid pus; thousands of little elevated bodies, variously discolored by venous congestion, thickly studded every portion of the peritoneum, being more thickly grouped over the liver and stomach, the organs that occasioned the greatest distress. The folds of the omentum and mesentery were loaded with them, and were greatly swelled and altered in texture. These tubercles were of various size—most commonly of the size of small shot or mustard seed, and were deposited in the subserous structure under the peritoneum. She had never complained of cough or pulmonary disease—but to satisfy myself of the non-implication of the lungs, I made several sections of them, but discovered no tubercles or other sign of pulmonary disease. The whole force of the malady seemed to have expended itself in the abdominal cavity. She was sick some nine or ten months—that is, from the period of cessation of her menstrual flow, the time from which she had dated her illness. All of my cases were young women, ranging from 18 to 21 or 22 years of age; and in all the very first complaint was a suppression of the menses, which never returned throughout the subsequent progress of the case.

At first the patient does not feel sick or make any complaint, but having missed one or two catamenial periods, she becomes concerned, and applies for something to restore the lost uterine function. After a longer or shorter interval, other symptoms come; such as a feeling of discomfort, wandering pains in the abdomen, a severe stitch in the side frequently recurring, a fit of nausea or vomiting, or diarrhœa alternating with constipation. The pain is not lancinating, or of that character that distinguishes ordinary peritoneal inflammation, and is unattended by fever, unless it happen to be accidental or an intercurrent one. It is such a pain as a hysterical or dyspeptic woman is apt to have, consequently it is likely not to occasion alarm. The appetite continues good, with occasional interruption by nausea or vomiting. The skin is natural. In a word, the patient does not think herself very sick, and attributes any discomfort she may feel to her menstrual irregularity. After a time, the abdomen swells, and finally presents unequivocal ascites. The case is worse than had been thought. There is obscure organic disease of grave import, but what organ is the sufferer? Attention is naturally turned to the liver, and diligent search is made in this locality. All the abdominal viscera are inquired after in turn, according to their importance, in the hope of finding the affected organ, but in vain. We reëxamine with still greater caution, till every gland, every function, every tissue whose derangement or disease might induce the result, however distant from the locality, have been questioned with the greatest caution and circumspection; yet with the same unrequited labor. There is no hepatic, renal, or cardiac disease that can be discovered; no history of an old peritonitis; no story of having "caught cold" by lying on damp ground—which means a sudden interruption of transpiration, the occasional cause of acute abdominal dropsy; whereas this affection of which I speak is chronic, and comes on without any assignable cause.

The ordinary remedies will generally remove the effusion, but fresh accumulations take place, and each successive time become more difficult of absorption. It continues to progress. Digestion becomes more interrupted; the nausea and vomiting more frequent; the abdominal uneasiness augments. The skin perhaps is dry, the tongue coated, the pulse quick, the urine scanty and high colored, the abdomen hot. If sudden pressure be made upon it, the recti muscles quickly contract, to ward off pressure from the underlying organs. Pain locates itself more intensely at a certain spot. To all appearances the case is now culminating. Inflammation has all at once revealed the hidden enemy: and what has been confused and tangled in the chain of morbid phenomena is suddenly understood and referred to inflammation of the liver, or of the stomach, or of the intestines, &c., &c., as the specialty of the symptoms may have determined.

The patient is now really, and is believed to be, dangerously ill. She is accordingly mercurialized, opiated, blistered, &c., as the symptoms may indicate or the emergency require. I do not think any one would bleed these cases. Presently the violence of the symptoms begins to abate, the fever to decline; the skins grow moist, the tongue clean, the urine clear and more abundant, and the bowels soluble; appetite returns, and the spirits rise. The patient begins to get strong, to sit up, to walk about; perhaps to attend to light household duties. But the rebound is not complete; the patient seemingly loiters on the threshold of health, and will advance no farther; the injury to the organism is too vast to admit of it. Some how, she wont get quite well. Presently there comes the miserable relapse. She complains of a pain here, or there, or there, till first one then another of the various abdominal viscera have been accused in turn of inflammation or other serious disorder; diarrhœa sets up its work of reduction, and what with deranged digestion and febrile irritation, the powers of the system are rapidly reduced —emaciation becoming daily more conspicuous.

If the hand be now laid on the abdomen it will be found intensely hot and drum-like; hectic is fairly set in, and this enemy allows the invalid no rest. But while life is thus hopelessly compromised and speedy death inevitable, yet, strange and incomprehensible, nature seems frequently to marshal her forces afresh and recover some of the lost ground. Thus, when daily expecting the death of the patient, we may hear of her getting better. But the effort is more spasmodic than formerly; if there was too much damage to important organs inflicted by the first attack of the disease to admit of recovery at that early stage of the malady, now more than ever is that end unattainable, as there has been fresh accessions of injury, embracing new organs and textures, perhaps equally necessary to the maintenance of life; therefore the counter-current soon sets in, till, finally worn out by combined hectic irritation and starvation, the patient passes out of life. Thus perished my first patient.

The second one died more quickly, seemingly destroyed by the violence of the excessive inflammation which the tubercles occasioned; she lived several months, and died suddenly of suppression of the urine, passing off in profound coma. For some time she had suffered from renal symptoms, discharging a small quantity of bloody urine, attended by considerable pain. A few days before the fatal issue, it stopped altogether. She was a colored girl, tall, good-looking, and of healthy appearance. A *most mortem* examination revealed myriads of miliary tubercles lining the cavity of the peritoneum. They literally covered the bladder and the kidneys, and were florid with the recent congestion. Very little adventitious membrane could be seen, but there was considerable bloody serum. The inflammatory action had not been sufficiently intense or prolonged for the evolution of this product. She died in the first stage. When it runs its full course, the various abdominal organs are bound, glued, and matted together by bands of this material, and very offensive pus may also be present. When the abdominal walls are fastened down upon the intestines or other viscera by adhesions, it may occasion tumors that give rise to great perplexity and error in the diagnosis. Having committed this mistake myself, I mention it to put others on their guard. It was the first case of this form of disease that came under my own observation, and on account of my inexperience was the source of more trouble to me than all of the rest combined. The patient was a young woman of 18 years. Her parents sent for me to ascertain the cause of her continued menstrual suppression—it was now over three months since her last catamenial flow. The abdomen was also swelled. The suppression of the menses and the abdominal swelling had come on without any cause she could assign. She had a healthy look; her pulse was natural, her skin comfortable, and her appetite good. I wont say what was my suspicion—but when I came to examine the abdomen, to my infinite surprise I found that it fluctuated. Beyond a question, there was fluid within the cavity of the peritoneum, and a little pains assured me it was not ovarian. The abdominal walls were uniformly distended, and the fluid shifted according as she changed her position. But how came it there? This was a problem difficult to solve. She had never suffered from peritonitis, or any kind of intestinal inflammation; there was no disease of the liver that could be discovered, nor of the heart, nor of the kidneys, nor had it resulted from a sudden chill, nor was there any anæmia to add its suggestion. Then how was it occasioned? To say that it resulted from some disturbance of the equilibrium between peritoneal secretion and absorption, was to make it as indefinite as ever, unless I could trace it to a disease of the peritoneum *per se;* this I could not do, as there had been no former peritonitis nor present tenderness; therefore I adopted what I then considered as the most plausible conclusion—that it resulted from some obstruction of the portal circulation; and whether this was hepatic or extra-hepatic I was forced to leave for future determination.

I prescribed brisk saline cathartics, alternating with the compound diuretic pill of squill, digitalis, and calomel. Some eight or ten days afterwards, I found considerable tympanitis, and whilst I was sitting by the bedside conducting my examination, her mother approached me, and asked "why her abdomen sounded so hollow, if it was full of water?" I told her that it had been removed, and gas had taken its place; that the medicine had acted well, and her daughter was much better. This was a most sagacious woman, and I therefore was careful not to excite her fears, unless I could satisfy her curiosity; but to be frank and honest, the tympanitic condition was even a deeper mystery to me than the dropsy had been. I could not understand it. It seemed to me to be a most novel case; but I did not feel alarmed for her safety, as her general condition was good, and there was very little abdominal uneasiness—not enough to cause suspicion of inflammatory action. She had occasionally felt nausea, and had vomited once or twice. The discharges from the bowels were still copious. Her tongue was a little furred. Her appetite had also suffered impairment, but there was no fever. She informed me that she was "very restless at night and could not sleep." I ordered a small pill, composed of equal parts of the mild chloride of mercury and opium, a half grain of each, morning, noon, and night; at the same time enjoining quiet, and simple but nutritious diet. She continued to improve, and was soon convalescent from her singular attack, as I believed, and I accordingly stopped visiting her. But I was mistaken, for she stopped short of complete recovery, and sent for me again, under a relapse, several weeks later. There had been a fresh accumulation of serous liquid in the cavity of the peritoneum. Of late she had suffered from irritable stomach, and was vomiting more than formerly. She was also constipated. There was considerable pain in the right hypochondrium, and pressure under the ribs greatly augmented it. At this point there was a feeling of distension, associated with deep throbbing pain. The pulse was excited, and she complained of thirst; the tongue was sharp, with red edges, and densely coated along its middle. I at last became fully satisfied that she was then laboring under acute hepatitis, and considering her former obscure symptoms, came to the conclusion that she had had the chronic or subacute form from the first, and it had betrayed itself by this sudden outburst or overt action; that somehow the ascitic collection was intimately associated with it, bearing the relationship of effect and cause. I accordingly treated her for acute hepatitis; I combated the most serious symptoms, and they were hepatic. She was many weeks in bed, and suffered much—had lost considerable flesh, and was broken down in body and mind, her face written over with the record of past suffering; she asked for articles of food fretfully, or with a sigh; she looked the confirmed invalid; she was better than she had been, but I dispared of her life. Wonderful to narrate, this patient began gradually and slowly the work of recovery. It was a long time before she walked her room with anything of ease or comfort. The serous collection, which had resisted every means made to dislodge it, suddenly disappeared of its own accord; so was it with other grave symptoms, one after an other departing as she continued to gain. She boasted of her appetite, of her returning strength, of her improvement generally. Alas! it was only a truce, for she very soon sent for me again, with worse symptoms than ever; this time she kept her bed till she died. Hectic now set in, to add to her debility and discomfort.

It was during this last phase of her malady that one day, whilst I was ascertaining the condition of her abdomen, I noticed a tumor in the right iliac region; it fluctuated, and was about the size of a cocoanut, containing perhaps a quart or more of liquid. No position in which I placed her succeeded in dislodging it. It was evidently sacculated. I could not resist the conclusion that it was ovarian dropsy, and I blamed myself for not having noticed it before. But when did it come on? This she did not know, but said "she had thought she had been swelled on that side for some time." Her abdomen was intensely hot, and pressure gave her pain; her stomach was very irritable, and she vomited often; severe diarrhœa alternated with constipation. The last symptom I had noticed throughout her entire illness, and I believe it a very valuable one, as it was also present in an equal or less degree in the other cases. One day I was vastly surprised, on examining the supposed ovarian tumor, to find that it contained gas instead of a liquid. I was truly amazed. A few weeks after this she died, when I discovered the phenomenon had had its origin in the formation of an adventitious cavity, caused by singular adhesions of intestines, bladder, omentum, and abdominal walls. It contained gas and a little bloody serum. Outside of this cavity, everything was bound, glued, and tied together in a confused mass, fœtid with pus. It beggared all description. Nothing escaped; not an organ or tissue in this cavity but showed marks of disease. The wonder was how she had managed so long to survive such ruin. The same answer would apply here as is given in cases of tuberculosis pulmonalis, where the organic structure of both lungs is nearly entirely destroyed—that the morbid change has been gradual, allowing the system opportunity to accommodate itself to each successive injury, thus in a manner becoming habituated to diseased action.

In conclusion, I will add, that whilst this affection is probably a form of scrofula, yet the cases that have come under my observation had not previously given any evidence of strumous cachexy; on the contrary, the patients were healthy and well developed, and consequently I was little prepared for such a denouement.

